# Supplementary material for: Distinct RPA domains promote recruitment and the helicase-nuclease activities of Dna2
Source: Nat Commun. 2021 Nov 11;12:6521. doi: 10.1038/s41467-021-26863-y (PMC8586334; doi:10.1038/s41467-021-26863-y)
Supplement: Supplementary file 1 — Supplementary Information [file 41467_2021_26863_MOESM1_ESM.pdf]

Distinct RPA domains promote recruitment and the helicase-nuclease activities of Dna2

Ananya Acharya, Kristina Kasaciunaite, Martin Göse, Vera Kissling, Raphaël Guérois, Ralf Seidel and Petr Cejka

Supplementary Information

Supplementary Figures 1-7
Supplementary Table S1 and S2

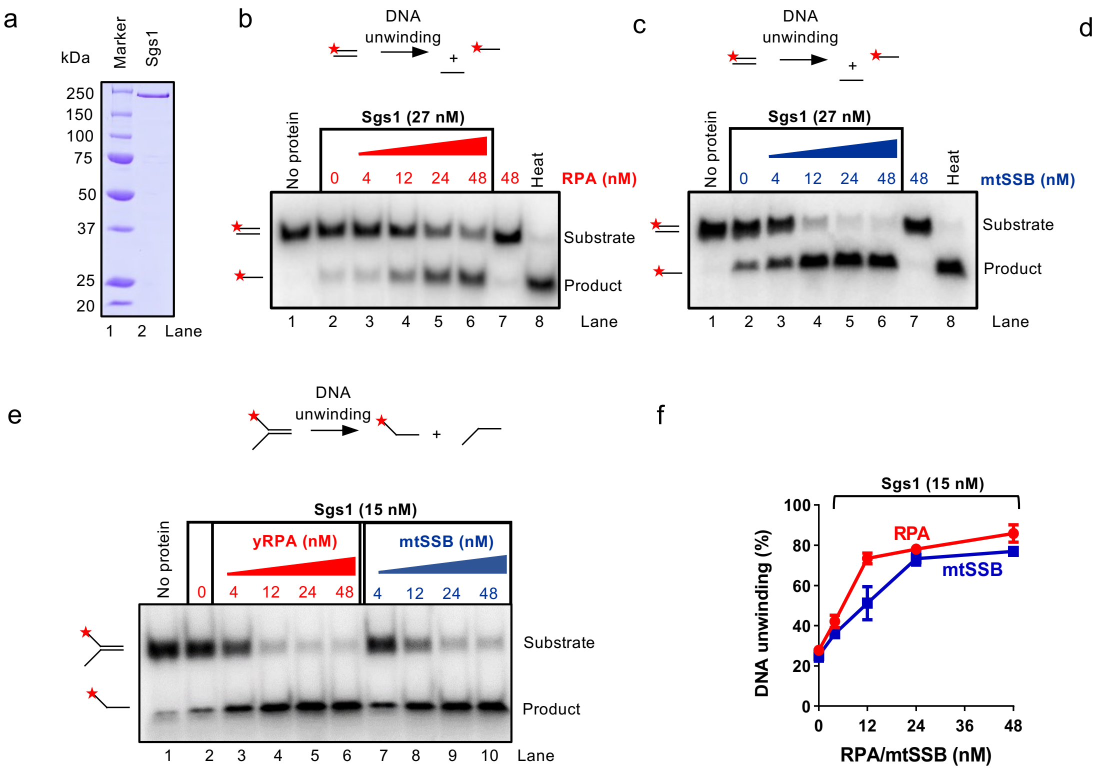

Supplementary Fig. 1 RPA does not specifically promote helicase and activity of Sgs1.

a Recombinant *S. cerevisiae* Sgs1 used in this study.

b-c Yeast RPA and mtSSB were used in helicase assays with Sgs1 and dsDNA substrate (50 bp, 1 nM, in molecules). The red asterisk indicates the position of the radioactive label.

d Quantification of assays such as shown in panels b and c. Error bars, SEM; n=3.

e Yeast RPA and mtSSB were used in helicase assays with Sgs1 and Y-shaped DNA substrate (25 nt ssDNA, 25 bp dsDNA, 1 nM, in molecules). The red asterisk indicates the position of the radioactive label.

f Quantification of assays such as shown in panel **e**. Error bars, SEM; n=3.

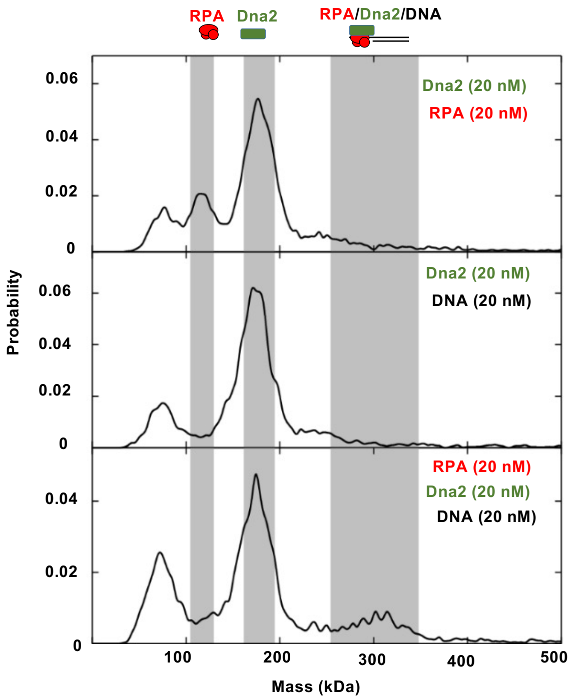

Supplementary Fig. 2 RPA stimulates Dna2 beyond recruitment to DNA.

a Measured molecular weight distributions of RPA and Dna2 complexes in absence and presence of a 5'-overhanged DNA (25 nt ssDNA, 48 bp dsDNA, 20 nM, in molecules). For the formation of a heterotrimeric complex (panel at the bottom), RPA was added first to the DNA followed by Dna2 addition.

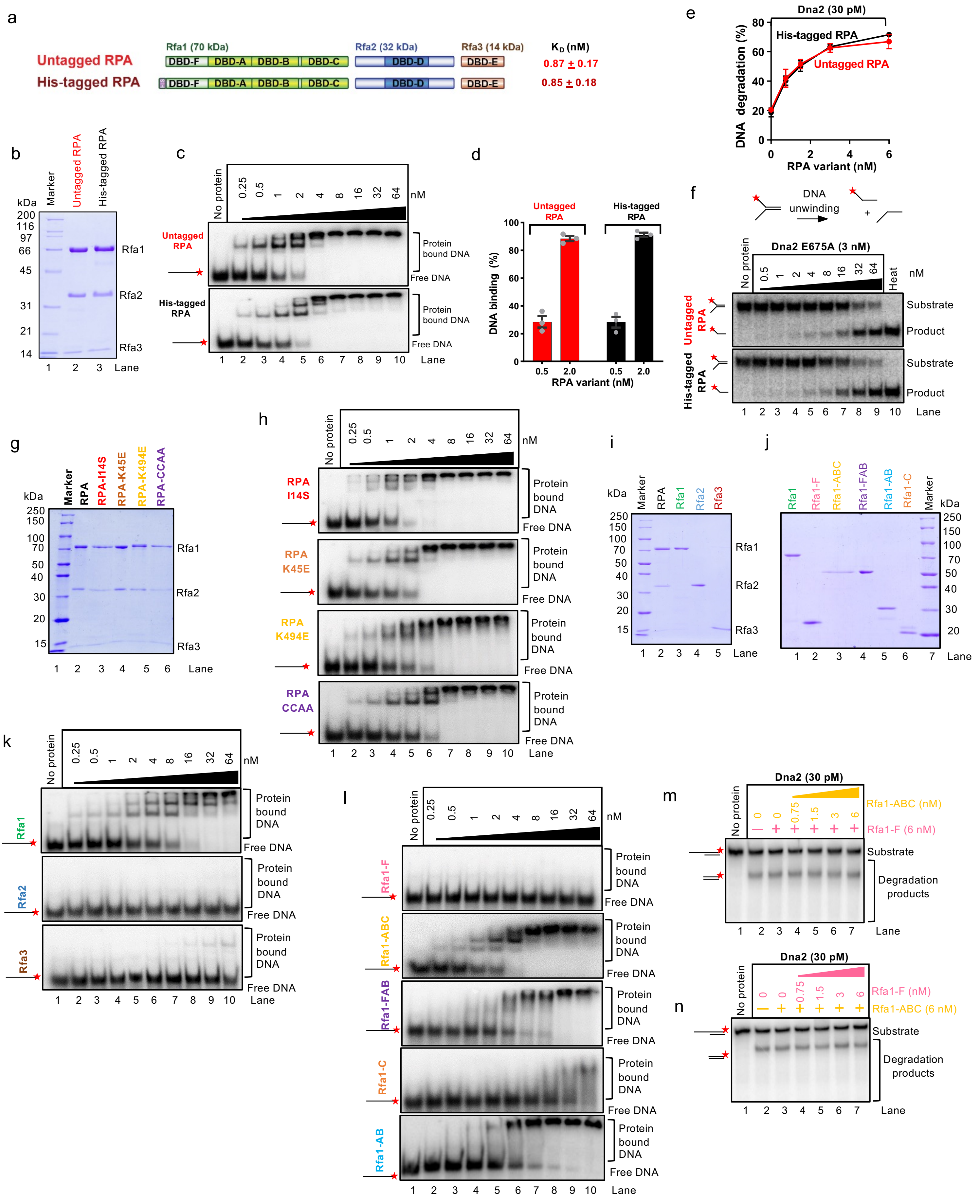

Supplementary Fig. 3 See next page for legend.

Supplementary Fig. 3 RPA-N terminus, as a part of the same polypeptide, is essential for the nuclease function of Dna2.

a A scheme of wild type untagged and his-tagged RPA. K_D, concentration of each RPA variant resulting in 50% binding to ssDNA (93 nt, 0.1 nM, in molecules) from experiments such as shown in panel c. Error, SEM; n=3.

b Wild type untagged and his-tagged RPA used in this study.

c Representative electrophoretic mobility shift assays to monitor DNA binding by untagged and his-tagged RPA to a ssDNA substrate (93 nt, 0.1 nM, in molecules). The red asterisk indicates the position of the radioactive label.

d Quantitation of ssDNA (93 nt, 0.1 nM, in molecules) binding by wild type untagged and his-tagged RPA as shown in panel c. His-tagged RPA is replotted as in Fig. 3b for comparison. Error bars, SEM; n=3.

e Quantification of nuclease assays using 5'-overhanged DNA substrate (45 nt ssDNA, 48 bp dsDNA, 1 nM, in molecules) with untagged and his-tagged RPA. Untagged RPA is replotted from Fig. 3c for comparison. Error bars, SEM; n=3.

f DNA unwinding of a forked DNA substrate (45 nt ssDNA, 48 bp dsDNA, 0.1 nM, in molecules) by Dna2-E675A in the presence of untagged or his-tagged RPA.

g His-tagged RPA point mutants used in this study.

h Representative electrophoretic mobility shift assays monitoring ssDNA binding (93 nt, 0.1 nM, in molecules) by RPA point mutants. One out of three independent experiments is shown.

i RPA subunits, expressed and purified individually, used in this study.

j Fragments of Rfa1 used in this study.

k Representative electrophoretic mobility shift assays to monitor ssDNA binding (93 nt, 0.1 nM, in molecules) by individual RPA subunits. One out of three independent experiments is shown.

l Representative electrophoretic mobility shift assay to monitor DNA binding by Rfa1 fragments to a ssDNA substrate (93 nt, 0.1 nM, in molecules). One out of three independent experiments is shown.

m Representative nuclease assays showing degradation of 5'-overhanged DNA substrate (45 nt ssDNA, 48 bp dsDNA, 1 nM, in molecules) using Dna2, Rfa1-F (6 nM) and increasing concentrations of Rfa1-ABC, as indicated. One out of three independent experiments is shown.

n Representative nuclease assay showing degradation of 5'-overhanged DNA substrate (45 nt ssDNA, 48 bp dsDNA, 1 nM, in molecules) using Dna2, Rfa1-ABC (6 nM) and increasing concentrations of Rfa1-F, as indicated. One out of three independent experiments is shown.

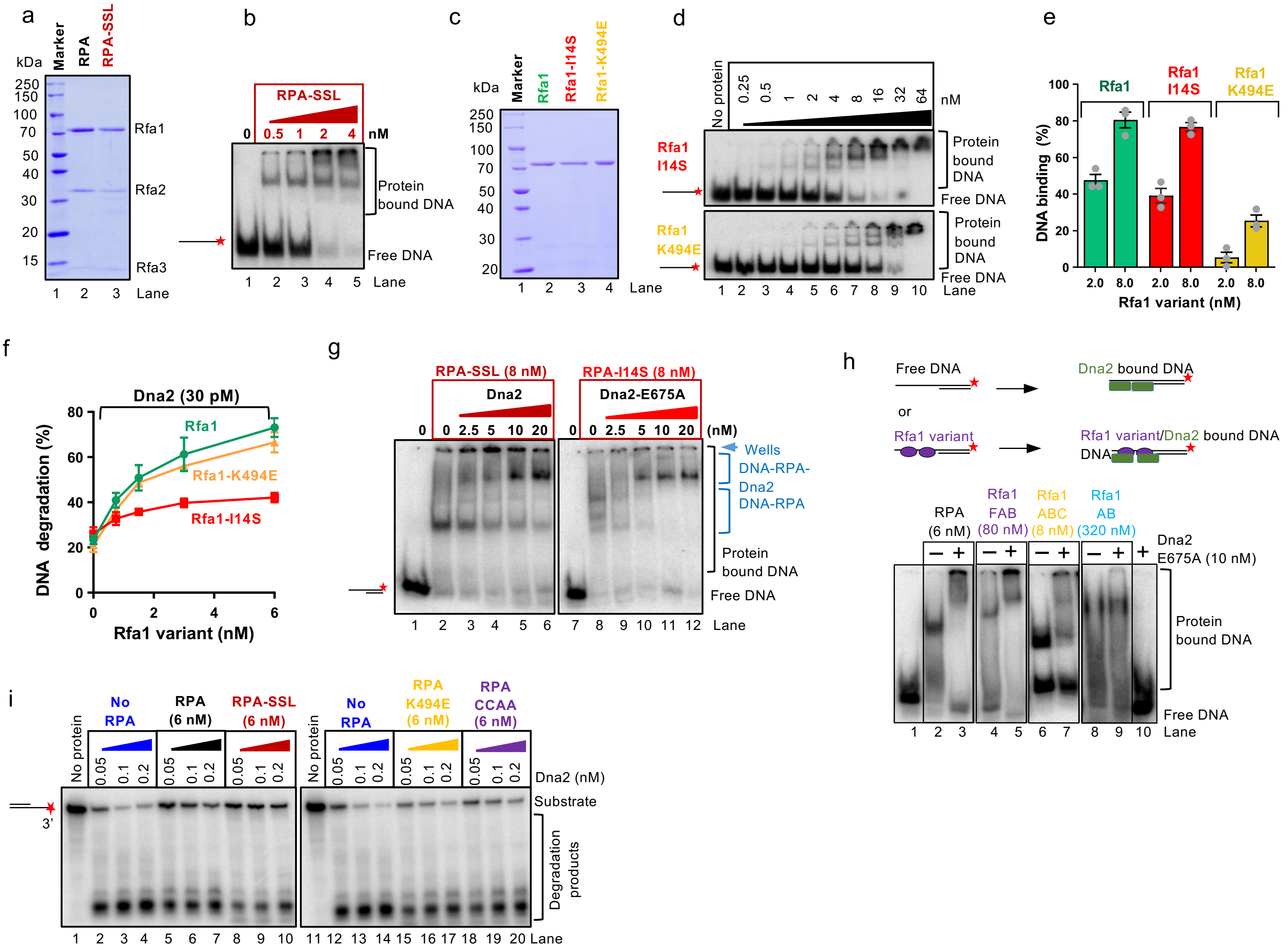

Supplementary Fig. 4 Dna2 recruitment, 5'-overhanged DNA degradation and 3'-overhanged DNA protection by yeast Dna2 require non-identical domains on RPA.

a Recombinant RPA and RPA-SSL (both his-tagged at N terminus of Rfa1) used in this study.

b Representative electrophoretic mobility shift assays to monitor ssDNA binding (93 nt, 0.1 nM, in molecules) by RPA-SSL. The red asterisk indicates the position of the radioactive label.

c Recombinant Rfa1 wild type and point mutants used in this study.

d Representative electrophoretic mobility shift assays to monitor ssDNA (93 nt, 0.1 nM, in molecules) binding by Rfa1 point mutants.

e Quantitation of ssDNA (93 nt, 0.1 nM, in molecules) binding by Rfa1 variants in assays such as shown in panel d. Rfa1 (2 nM) is replotted from Fig. 3e for reference. Error bars, SEM; n=3.

f Quantification of nuclease assays with Rfa1 variants. Rfa1 is replotted from Fig. 3g for reference. Error bars, SEM; n=3.

g Representative electrophoretic mobility shift assays to monitor recruitment of Dna2 or Dna2-E675A to either RPA-SSL or RPA-I14S-precoated (8 nM) 5'-overhanged DNA substrate (45 nt ssDNA, 48 bp dsDNA, 1 nM, in molecules) in the presence of 3 mM EDTA (lanes 1-6) or 5 mM magnesium acetate (lanes 7-12), respectively, and 150 mM NaCl. One out of two independent experiments is shown.

h Representative electrophoretic mobility shift assays to monitor recruitment of Dna2-E675A to 5'-overhanged DNA substrate (30 nt ssDNA, 31 bp dsDNA, 1 nM, in molecules), precoated with the respective RPA variant, in the presence of 5 mM Mg^{2+} and 150 mM NaCl. One out of three independent experiments is shown.

i Representative nuclease assays showing protection of 3'-overhanged DNA substrate (45 nt ssDNA, 48 bp dsDNA, 1 nM, in molecules) from Dna2 degradation by the respective RPA variants. Assays contained 100 mM NaCl. One out of four independent experiments is shown.

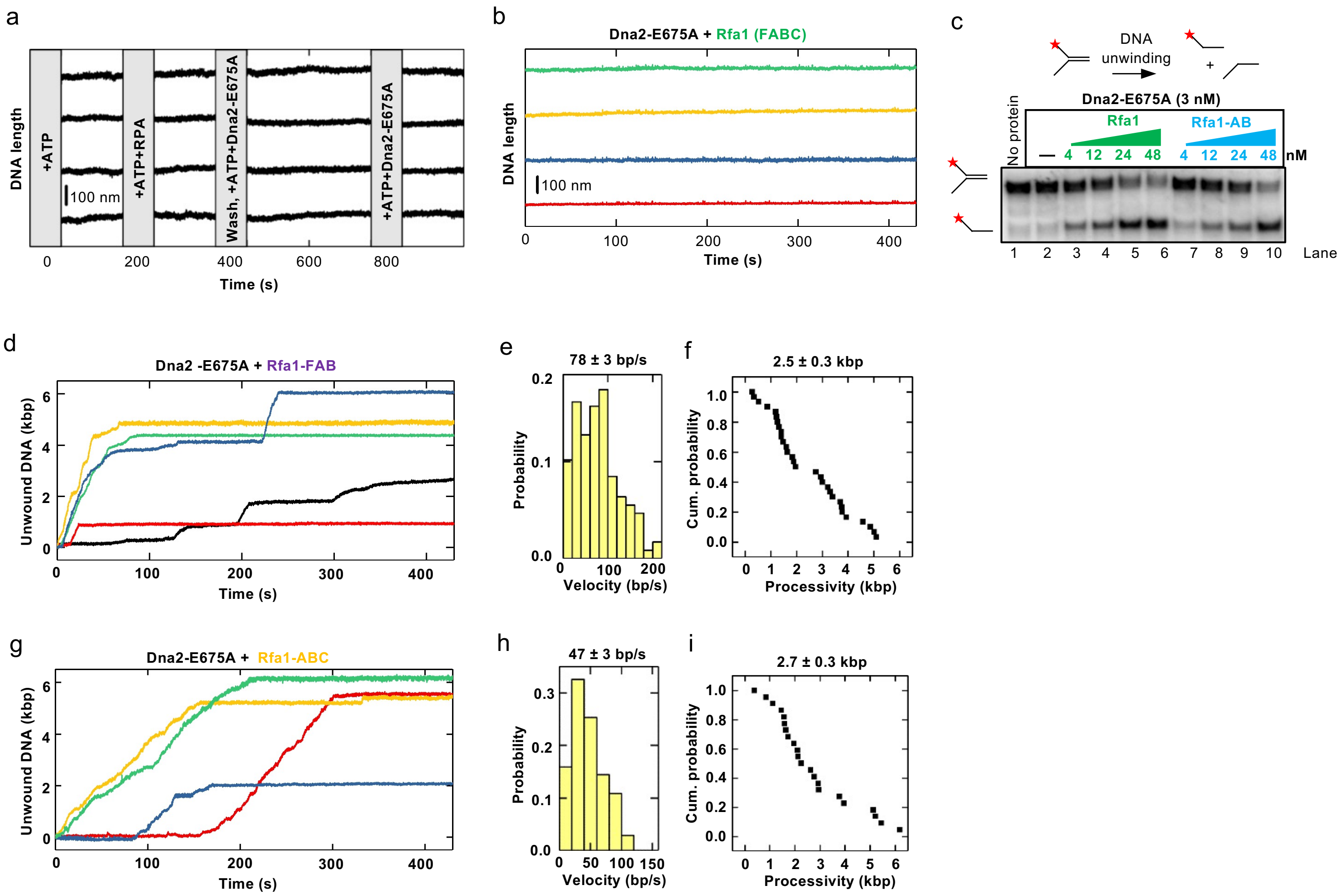

Supplementary Fig. 5 DNA degradation and DNA unwinding by yeast Dna2 require different domains on RPA.

a RPA is required for DNA unwinding by Dna2-E675A. The flowcell was supplemented with 2 nM RPA to bind and saturate the overhangs of the DNA construct. After 10 min, the flowcell was slowly washed with 2 ml buffer to remove unbound RPA molecules. Subsequently, Dna2-E675A (5 nM) was added. After 400 s, fresh Dna2-E675A (5 nM) was supplied. No unwinding events were observed, suggesting that auxiliary RPA is needed to support progressing unwinding beyond the initiation of the reaction.

b Rfa1 does not promote Dna2-E675A helicase activity. DNA unwinding was monitored in the presence of Dna2-E675A (5 nM) and Rfa1 (20 nM).

c DNA unwinding of a forked DNA substrate (45 nt ssDNA, 48 bp dsDNA, 0.1 nM, in molecules) by Dna2-E675A in the presence of Rfa1 and Rfa1-AB with 50 mM KCl. The red asterisk indicates the position of the radioactive label.

d Representative DNA unwinding events catalyzed by Dna2-E675A (5 nM) in the presence of Rfa1-FAB (20 nM).

e Histogram of the unwinding rates (n=22) from experiments such as shown in panel d. Error, SEM.

f Cumulative probability distribution (shown as survival probability) of the processivity of the individual unwinding events (n=22) from experiments such as shown in panel d. Error, SEM.

g Representative DNA unwinding events catalyzed by Dna2-E675A (5 nM) in the presence of Rfa1-ABC (20 nM).

h Histogram of the unwinding rates (n=22) from experiments such as shown in panel g. Error, SEM.

i Cumulative probability distribution (shown as survival probability) of the processivity of the individual unwinding events (n=22) from experiments such as shown in panel g. Error, SEM.

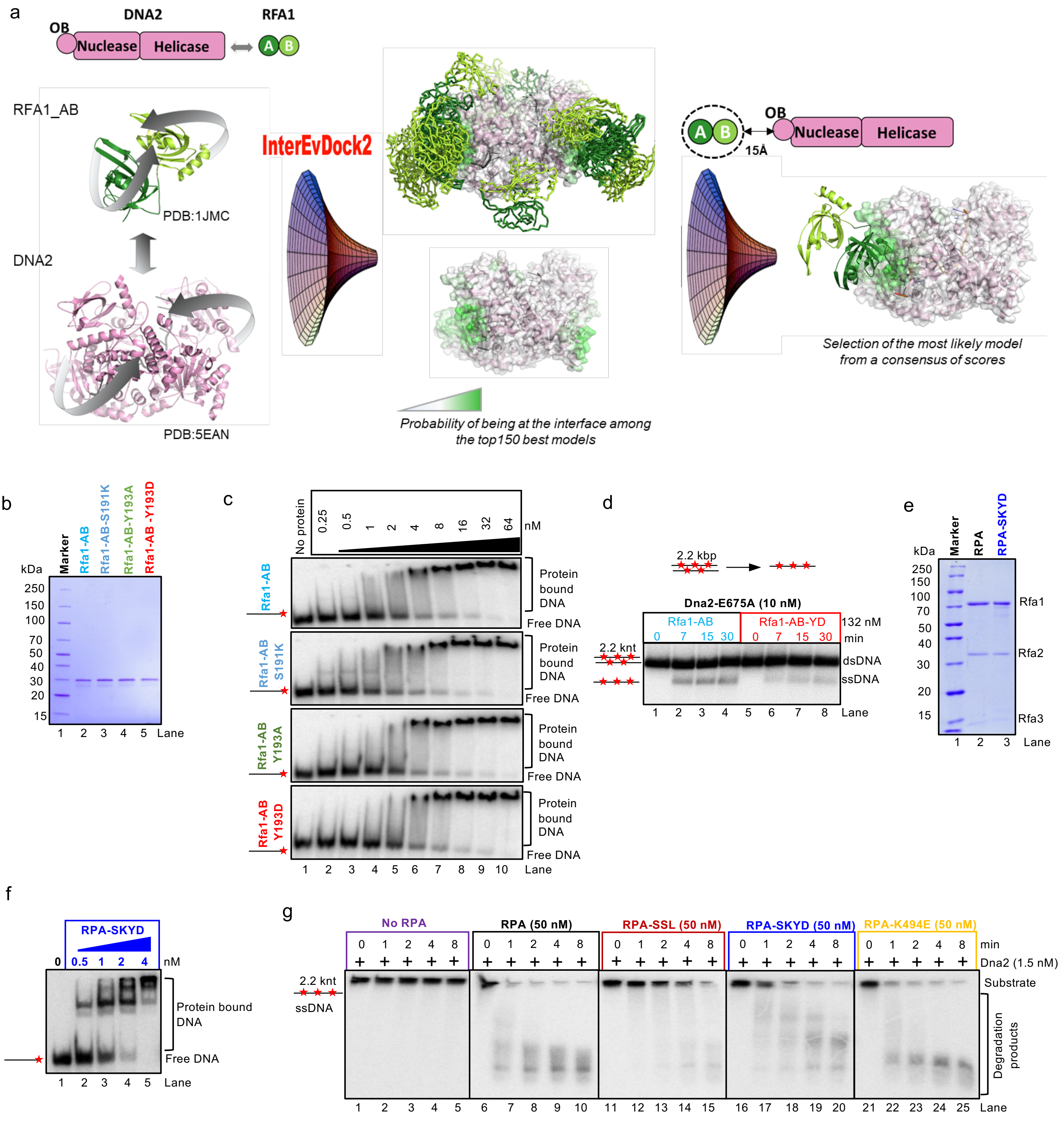

Supplementary Fig. 6 RPA-DBDA and B constitute the minimal RPA domain required for catalyzing Dna2 unwinding function.

a Schematic pipeline used for the docking simulation between the structures of human RFA1_AB domains (green cartoon) and of human DNA2 (pink cartoon). After a global rigid-body search using the InterEvDock2 server, a consensus of 150 best models were recovered showing that the most likely solutions tended to cluster in two major regions at the surface of DNA2. The green gradient at the surface of DNA2 molecule indicates the regions most frequently involved in the interface with the RFA1_AB domains. In a second step, the docking simulation was repeated increasing the sampling in the region located near the location of the ssDNA 3' end thanks to a 15A constraint applied between residues found to interact in the initial global docking simulation. This second step led to a set of consistent solutions which could be selected for further refinement steps as described in Methods section.

Continued on next page.

Continued from previous page.

b Recombinant wild type Rfa1-AB and point mutants used in this study.

c Representative electrophoretic mobility shift assays to monitor ssDNA (93 nt, 0.1 nM, in molecules) binding by wild type Rfa1-AB and point mutants. The red asterisk indicates the position of the radioactive label. One out of three independent experiments is shown.

d Representative experiments showing kinetics of 2.2 kbp-long dsDNA (0.1 nM, in molecules) unwinding by Dna2-E675A in the presence of Rfa1-AB and Rfa1-AB-Y193D. The experiment was performed three times with similar results. One out of three independent experiments is shown.

e Recombinant wild type RPA and RPA-SKYD used in this study.

f Representative electrophoretic mobility shift assays to monitor ssDNA (93 nt, 0.1 nM, in molecules) binding by RPA-SKYD point mutant. One out of three independent experiments is shown.

g Representative nuclease assays showing kinetics of 2.2 knt-long ssDNA (0.3 nM, in molecules) degradation by Dna2 in the presence of RPA variants. One out of three independent experiments is shown.

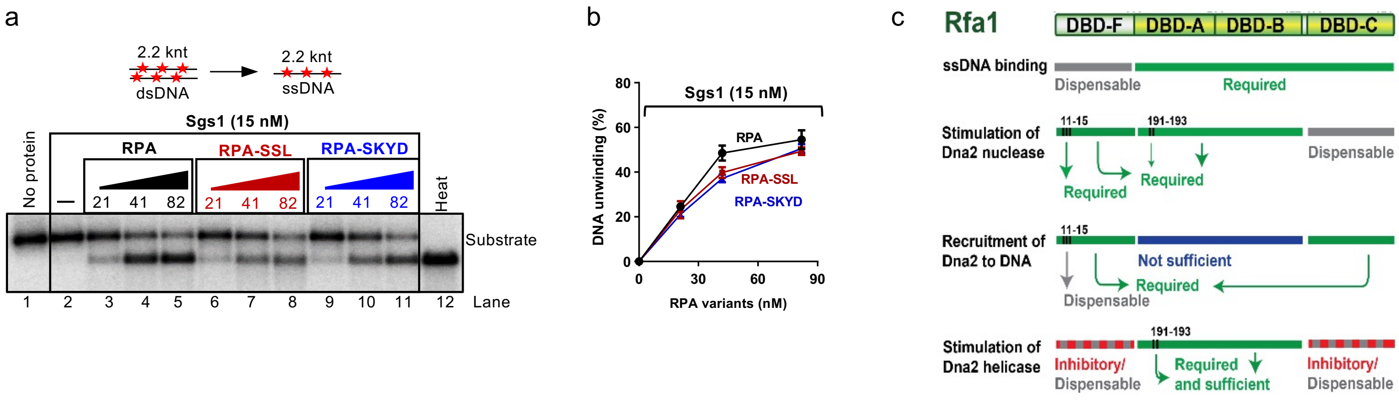

Supplementary Fig. 7. RPA domains involved in Dna2 stimulation.

a Representative experiments showing unwinding of 2.2 kbp-long dsDNA (0.1 nM, in molecules) by Sgs1 in the presence of Rfa1-AB variants. Red asterisks indicate random radioactive labels on the DNA.

b Quantification of assays such as shown in panel a. Error bars, SEM; n=3.

c A schematic representation of the various Rfa1 domains involved in ssDNA binding, recruitment of Dna2 to DNA, and Dna2 nuclease and helicase stimulation.

Table S1. List of oligonucleotides used for cloning and site-directed mutagenesis in this study.

No.	Construct	Forward Primer (FP)	Reverse Primer (RP)
1	p11d-his-sctRPA	AA07	AA08
2	p11d-his-sctRPA-I14S	AA13	AA14
3	p11d-his-sctRPA-K45E	AA15	AA16
4	p11d-his-sctRPA-K494E	AA17	AA18
5	p11d-his-sctRPA-CCAA	AA19	AA20
6	p11d-his-sctRPA-SSL	yeast RPA1 triple mutant FP	yeast RPA1 triple mutant RP
7	p11d-his-sctRPA-SKYD	Rfa1-SK-YD-FP	Rfa1-SK-YD-RP
8	pET21-T7-Rfa1-His	AA01	AA02
9	pET21-T7-Rfa2-His	AA03	AA04
10	pET21-T7-Rfa3-His	AA05	AA06
11	pET21-T7-Rfa1-F-His	AA01	AA09
12	pET21-T7-Rfa1-FAB-His	AA01	AA11
13	pET21-T7-Rfa1-AB-His	AA10	AA11
14	pET21-T7-Rfa1-ABC-His	AA10	AA02
15	pET21-T7-Rfa1-C-His	AA12	AA02
16	pET21-T7-Rfa1-I14S-His	AA13	AA14
17	pET21-T7-Rfa1-K494E -His	AA17	AA18
18	pET21-T7-Rfa1-AB-S191K-His	Rfa1- S191K FP	Rfa1- S191K RP
19	pET21-T7-Rfa1-AB-Y193A-His	Rfa1-Y193A FP	Rfa1-Y193A RP
20	pET21-T7-Rfa1-AB-Y193D-His	Rfa1- Y193D FP	Rfa1- Y193D RP

Table S2. Sequence of oligonucleotides used for cloning and site-directed mutagenesis in this study.

No.	Name	Sequence (5'-3')	Purpose
1	AA01	CCTGCCGGATCCGGCAGTGTTCAACTTTCGAGGGGC	Rfa1 FP
2	AA02	CCTGCCCTCGAGAGCTAACAAAGCCTTGGATAAC	Rfa1 RP
3	AA03	CCTGCCGGATCCGCAAGTTATCAACCATATAACG	Rfa2 FP
4	AA04	CCTGCCCTCGAGTAGGGCAAAGAAGTTATTGTC	Rfa2 RP
5	AA05	CCTGCCGGATCCGCCAGCGAAACACCAAGAGTTG	Rfa3 FP
6	AA06	CCTGCCCTCGAGGTATATTTCTGGGTATTTCTTAC	Rfa3 RP
7	AA07	CATGCACCATCACCATCACCAT	6x His FP
8	AA08	CATGATGGTGATGGTGATGGTG	6x His RP
9	AA09	CCTGCCCTCGAGTTTTTGCGAATTAGGGTTTTC	Rfa1 1-180 RP
10	AA10	CCTGCCGGATCCACCAGACCAATTTTTGCCATCG	Rfa1 181-621 FP
11	AA11	CCTGCCCTCGAGTTTTGTTAAGCTAGCAGCCGATTG	Rfa1 1-442 RP
12	AA12	CCTGCCGGATCCTTCATTGCTCAGCGTATTAC	Rfa1 443-621 FP
13	AA13	GGGGCGATTTTCATAGCAGCTTCACCAATAAGCAAAGG	Rfa1- I14S FP
14	AA14	CCTTTGCTTATTGGTGAAGCTGCTATGAAAATCGCCCC	Rfa1- I14S RP
15	AA15	GGGGCTAACAGCAACAGAGAGAATTTGATCATGATTTCC	Rfa1-K45E FP
16	AA16	GGAAATCATGATCAAATTCTCTCTGTTGCTGTTAGCCCC	Rfa-K45E RP
17	AA17	CTAATGAGAATTGTAATAAG GAA GTT CTG GAA CAG CCT GATGG	Rfa1-K494E FP
18	AA18	CAGGCTGTTCCAGAACTTCCTTATTACAATTCTCATTAG	Rfa1-K494E RP
19	AA19	CCTGATGGTACTTGGAGAGCTGAGAAGGCCGACACCAATAATGCAAGG	Rfa1-CCAA FP
20	AA20	CCTTGCATTATTGGTGTCGCCTTCTCAGCTCTCCAAGTACCATCAGG	Rfa1-CCAA RP
21	yeast RPA1 triple mutant FP	GGGGCGATTCTCATAGCAGCCTCACCAATAAGCAAAGGTACG	Rfa1-SSL-FP
22	yeast RPA1 triple mutant RP	CGTACCTTTGCTTATTGGTGAGGCTGCTATGAGAATCGCCCC	Rfa1-SSL-RP
23	Rfa1-S191K FP	CCAATTTTTGCCATCGAACAACTGAAGCCATACCAAAACGTTTGGACTATC	Rfa1-S191K FP
24	Rfa1-S191K RP	GATAGTCCAAACGTTTTGGTATGGCTTCAGTTGTTCGATGGCAAAAATTGG	Rfa1-S191K RP
25	Rfa1-Y193A FP	GCCATCGAACAACTGTCTCCAGCCCAAAACGTTTGGACTATC	Rfa1-Y193A FP
26	Rfa1-Y193A RP	GATAGTCCAAACGTTTTGGGCTGGAGACAGTTGTTCGATGGC	Rfa1-Y193A RP
27	Rfa1-Y193D FP	GCCATCGAACAACTGTCTCCAGACCAAAACGTTTGGACTATC	Rfa1-Y193D FP
28	Rfa1-Y193D RP	GATAGTCCAAACGTTTTGGTCTGGAGACAGTTGTTCGATGGC	Rfa1-Y193D RP
29	Rfa1-SK-YD-FP	CCAATTTTTGCCATCGAACAACTGAAGCCAGACCAAAACGTTTGGACTATCAAAGCAAG	RPA-SKYD FP
30	Rfa1-SK-YD-RP	CTTGCTTTGATAGTCCAAACGTTTTGGTCTGGCTTCAGTTGTTCGATGGCAAAAATTGG	RPA-SKYD RP
