# Supplementary material for: Distinct RPA domains promote recruitment and the helicase-nuclease activities of Dna2
Source: Nat Commun. 2021 Nov 11;12:6521. doi: 10.1038/s41467-021-26863-y (PMC8586334; doi:10.1038/s41467-021-26863-y)
Supplement: Supplementary file 3 — Reporting Summary [file 41467_2021_26863_MOESM3_ESM.pdf]

nature portfolio

Corresponding author(s): Petr Cejka

Last updated by author(s): Sep 22, 2021

Reporting Summary

Nature Portfolio wishes to improve the reproducibility of the work that we publish. This form provides structure for consistency and transparency in reporting. For further information on Nature Portfolio policies, see our Editorial Policies and the Editorial Policy Checklist.

Statistics

For all statistical analyses, confirm that the following items are present in the figure legend, table legend, main text, or Methods section.

n/a	Confirmed	
☐	☒	The exact sample size (*n*) for each experimental group/condition, given as a discrete number and unit of measurement
☐	☒	A statement on whether measurements were taken from distinct samples or whether the same sample was measured repeatedly
☒	☐	The statistical test(s) used AND whether they are one- or two-sided<br>*Only common tests should be described solely by name; describe more complex techniques in the Methods section.*
☒	☐	A description of all covariates tested
☒	☐	A description of any assumptions or corrections, such as tests of normality and adjustment for multiple comparisons
☐	☒	A full description of the statistical parameters including central tendency (e.g. means) or other basic estimates (e.g. regression coefficient) AND variation (e.g. standard deviation) or associated estimates of uncertainty (e.g. confidence intervals)
☒	☐	For null hypothesis testing, the test statistic (e.g. *F*, *t*, *r*) with confidence intervals, effect sizes, degrees of freedom and *P* value noted<br>*Give P values as exact values whenever suitable.*
☒	☐	For Bayesian analysis, information on the choice of priors and Markov chain Monte Carlo settings
☒	☐	For hierarchical and complex designs, identification of the appropriate level for tests and full reporting of outcomes
☒	☐	Estimates of effect sizes (e.g. Cohen's *d*, Pearson's *r*), indicating how they were calculated

Our web collection on statistics for biologists contains articles on many of the points above.

Software and code

Policy information about availability of computer code

Data collection	Scans of radioactive gels were acquired using TyphoonTM FLA 9500 software, version 1.0 and the kinetic ATPase assay data were collected using the software Cary WinUV Kinetics Application, version 5.0.0.999. Magnetic tweezers were operated using a self-developed code in Labview (2016, National Instruments). AcquireMP (Refeyn Ltd, Version 2.3.0) was used for mass photometry data collection.
Data analysis	The radioactive gels in this study were analyzed using ImageJ 1.53g and the results were plotted using GraphPad Prism 9.0.2. Magnetic tweezers trajectories were analyzed using a custom-code written in MATLAB (2016a). See https://doi.org/10.5281/zenodo.5524562 for the code.<br>Data analysis as well as plotting of magnetic tweezers and mass photometry data was carried out in Origin 2017G.<br>DiscoverMP (Refeyn Ltd, version 2.3.0) was used for mass photometry data analysis.<br>Software used to generate the model of the docking assemblies was: For the rigid-body docking section: InterEvDock, version 2, http://bioserv.rpbs.univ-paris-diderot.fr/services/InterEvDock2/<br>For the comparative modeling and for relaxing the models: Rosetta, version rosetta_src_2017.08.59291, https://www.rosettacommons.org/<br>Multiple Sequence Alignments were represented using JalView version 2.11.1.4, and generated using mafft algorithm, version 7.307.

For manuscripts utilizing custom algorithms or software that are central to the research but not yet described in published literature, software must be made available to editors and reviewers. We strongly encourage code deposition in a community repository (e.g. GitHub). See the Nature Portfolio guidelines for submitting code & software for further information.

Data

Policy information about availability of data

All manuscripts must include a data availability statement. This statement should provide the following information, where applicable:

- Accession codes, unique identifiers, or web links for publicly available datasets
- A description of any restrictions on data availability
- For clinical datasets or third party data, please ensure that the statement adheres to our policy

Relevant data generated or analyzed during this study are included in this article and its supple-mentary information. Source data are provided with this paper, and in a repository: https://doi.org/10.5281/zenodo.5524562. The structural models data were deposited in the ModelArchive database and are available: https://modelarchive.org/doi/10.5452/ma-q8w8e, with the following password: c9z33sysJh. Requests for material will be fulfilled by the corresponding author.

Field-specific reporting

Please select the one below that is the best fit for your research. If you are not sure, read the appropriate sections before making your selection.

[x] Life sciences [ ] Behavioural & social sciences [ ] Ecological, evolutionary & environmental sciences

For a reference copy of the document with all sections, see nature.com/documents/nr-reporting-summary-flat.pdf

Life sciences study design

All studies must disclose on these points even when the disclosure is negative.

Sample size	Sample size (or number of repeats) was chosen based on what is common in the field, and what was practical to do.
Data exclusions	In general, no data were excluded unless there was a valid reason to do so, e.g. experiments with failed positive controls indicating technical problems, or when loading control indicated unequal loading that invalidated the analysis or other technical issues (broken gels, collapsed wells in gels etc.).
Replication	The experiments were replicated several times as indicated in figure legends. Oftentimes, even within a single experiment, multiple enzyme concentrations were analyzed, or samples were compared at multiple time points. This also contributes to the robustness of the data.
Randomization	Randomization is not relevant to the experiments performed in this study.
Blinding	Blinding is not relevant to the experiments in this study, as measurements were objectively quantified by dedicated software or simply visually presented. Furthermore, the loading order of samples on gels prevented blinding.

Reporting for specific materials, systems and methods

We require information from authors about some types of materials, experimental systems and methods used in many studies. Here, indicate whether each material, system or method listed is relevant to your study. If you are not sure if a list item applies to your research, read the appropriate section before selecting a response.

Materials & experimental systems

n/a	Involved in the study	
[x]	[ ]	Antibodies
[ ]	[x]	Eukaryotic cell lines
[x]	[ ]	Palaeontology and archaeology
[x]	[ ]	Animals and other organisms
[x]	[ ]	Human research participants
[x]	[ ]	Clinical data
[x]	[ ]	Dual use research of concern

Methods

n/a	Involved in the study	
[x]	[ ]	ChIP-seq
[x]	[ ]	Flow cytometry
[x]	[ ]	MRI-based neuroimaging

Eukaryotic cell lines

Policy information about cell lines

Cell line source(s)	We used Sf9 cells adapted for suspension growth, available from the cell line collection of the Institute of Molecular Cancer Research, University of Zurich.
Authentication	The cell line was not authenticated.
Mycoplasma contamination	The cell line was not tested for mycoplasma contamination.
Commonly misidentified lines (See ICLAC register)	No misidentified cell lines were used.
